# Factors Influencing the Length of Hospital Stay Among Pediatric COVID-19 Patients at Queen Rania Al Abdullah Hospital for Children: A Cross-Sectional Study

**DOI:** 10.7759/cureus.35000

**Published:** 2023-02-14

**Authors:** Alia M Al-Khlaifat, Asmaa M Al Quraan, Aseel F Nimri, Nasser Eyadeh Bani Khaled, Nusaeibah Ramadina, Fadi F Ayyash, Shadi O Daoud, Sarah Y Hamlan, Bayan M Hababeh

**Affiliations:** 1 Pediatric Infectious Diseases, Jordanian Royal Medical Services, Amman, JOR; 2 Research, California Institute of Behavioral Neurosciences & Psychology, Fairfield, USA; 3 Pediatric Medicine, Jordanian Royal Medical Services, Amman, JOR; 4 Pediatric Endocrinology, Jordanian Royal Medical Services, Amman, JOR; 5 Rheumatology, Jordanian Royal Medical Services, Amman, JOR; 6 Pediatrics, Jordanian Royal Medical Services, Amman, JOR

**Keywords:** budget management, healthcare insurance, use of steroids, respiratory signs, respiratory symptoms, comorbidity, children, length of stay

## Abstract

Background

COVID-19 caused by SARS-CoV-2 is a worldwide epidemic. Children are less commonly infected and have less severe symptoms than adults. However, they are at risk for COVID-19-associated severe sickness and hospitalization. The duration of stay is a major driver of effective health treatment during hospitalization; thus, it is only logical to attempt to comprehend the factors influencing the length of stay (LOS) for these patients, particularly in light of the ongoing pandemic caused by the new SARS-CoV-2 virus. As predictors of hospital LOS, several variables, including age, gender, disease severity, hospital mortality, insurance type, and hospital location, have been discovered. In our study, we focused on the severity of the patient's condition, the presence of comorbidities, and the necessary therapeutic regimen to predict the duration of stay. This study aimed to answer the following questions: If a patient has comorbidity and has COVID-19 requiring hospital treatment, will the patient's comorbidity elongate the duration of stay at the hospital for further management in the pediatric age group? What are the risk factors that play a significant role in the hospital stay duration in pediatrics?

Methodology

We gathered data from 100 hospitalized children aged up to 14 years who tested positive for COVID-19, which was not specific to variants of SARS-CoV-2, over 24 months (February 2020-February 2022) at Queen Rania Al Abdullah Hospital for Children, one of the Health Care Accreditation Council accredited facilities. Clinical symptoms, signs, oxygen demand, imaging study results, laboratory data, and usage of corticosteroid and antiviral medication were all taken from patients' medical records. There were no limitations in taking the sample of patients. All patients in the duration mentioned were included.

Results

Clinical data of 100 COVID-19-positive pediatric patients were analyzed; 52% of the patients had associated chronic illnesses, while 48% were medically free. The longest duration of LOS was 28 days, the shortest was one day, the median was eight days, and five days was the most frequent among patients owing to 21% of patients, using mean descriptive statistics. We compared LOS to having or not having comorbidities. The mean LOS of patients with the comorbid disease was 6.15 days, with a maximum of 28 days, while for patients without chronic illnesses, the mean was 4.81 days with a maximum of 14 days. The significance was 0.07. Our results also showed a significant correlation between using steroids and LOS, as it had an advantageous effect by decreasing it with a significance value of 0.04. Having abnormal findings on chest computed tomography (CT) scan was also associated with increased LOS with a significant value of 0.00.

Conclusions

According to our research, there was no direct association between comorbidity and hospital LOS, which is counterintuitive, as it was influenced by multiplayers of variables such as using steroids, which decreased the LOS, and abnormal findings on chest CT, which resulted in lengthening of the hospital stay. Our findings cannot be proven without further research and a larger patient sample.

## Introduction

COVID-19 appeared in Wuhan, China, in November 2019. It quickly became a worldwide pandemic that caused 62.2 million illnesses and 1.4 million deaths by 2020. Initial symptoms of COVID-19 might occur three days after exposure. Some of the most prevalent symptoms include fever, tiredness, and anosmia. Presymptomatic transmission is thought to be a major contributor to the propagation of the virus. The condition ranges from asymptomatic to acute respiratory distress syndrome. Although the fatality rate is still disputed, it is thought to be lower than other respiratory infections such as SARS-CoV-2 and Middle East respiratory syndrome (MERS) as 5% of patients exhibit symptoms that necessitate hospitalization and the mortality rate ranges from 5% to 25% [1].

Like other viruses, SARS-CoV-2 evolves. The vast majority of SARS-CoV-2 genetic changes do not affect viral function. Variants of concern are variations that have garnered significant interest due to their rapid appearance in populations and evidence of transmission or major clinical effects. Each variant has many names based on the terminology used by various evolutionary classification systems. The World Health Organization (WHO), however, has assigned Greek alphabet labels to notable variants [2]. Currently, Omicron is the variant of concern. In November 2021, this variation was first found in Botswana and afterward in South Africa. Omicron accounted for the bulk of new infections in the United States as of late December 2021. It was connected to an increase in regional infections in South Africa and was rapidly discovered in several other nations. Emerging research on Omicron's clinical impact reveals that it is more transmissible than the Delta variant and more resistant to infection and vaccine-induced humoral immunity but associated with milder symptoms [3]. The Delta variant was identified in India in December 2020, and before the appearance of the Omicron variant, it was the most prevalent type globally. The Delta variant was more transmissible than the Alpha version and was associated with a higher risk of hospitalization and serious illness. Several studies indicate that the effectiveness of an immunization against symptomatic Delta infection is greatly diminished, although it remains high against severe disease and hospitalization [4]. The Alpha variant was found in the United Kingdom in late 2020 and became the dominant variant globally until the emergence of the Delta variant. The Alpha variant was 50% to 75% more contagious than previously circulating viruses. According to specific studies, the Alpha variant was associated with more severe disease [5].

Patients infected with SARS-CoV-2 can have a variety of clinical presentations, ranging from no symptoms to serious disease [6]. The disease ranges as follows, asymptomatic or presymptomatic infection in which individuals test positive for SARS-CoV-2 using a virologic test (i.e., a nucleic acid amplification test or an antigen test) but have no characteristic symptoms of COVID-19. Individuals with mild illness exhibit many of the symptoms and clinical signs of COVID-19 as fever, sore throat, cough, fatigue, headache, myalgia, nausea, vomiting, diarrhea, and diminished taste, and smell sensations but do not exhibit shortness of breath or abnormal findings on chest radiographs. Individuals with moderate illness, on the other hand, have a saturation of oxygen (SpO2) of 94% on room air at sea level and exhibit indications of lower respiratory disease during clinical examination or imaging. Furthermore, individuals with an SpO2 of 94% on room air at sea level, a ratio of arterial partial pressure of oxygen to fraction of inspired oxygen (PaO2/FiO2) of 300 mmHg, a respiratory frequency of more than 30 breaths per minute, or lung infiltrates of more than 50% are considered to be in severe illness. Finally, patients with severe respiratory failure, septic shock, and/or multiple organ dysfunction are considered to be in critical condition [7]. Children often have a less severe version of the disease than adults. Globally, infants and neonates infected with COVID-19 have been recorded. To offer correct information to neonatologists and pediatricians, it is crucial to accurately define the clinical manifestations and therapy of COVID-19 infection in this age group [8]. It was considered that the majority of COVID-19 cases in children were asymptomatic or exhibited little clinical signs. However, the situation appears to be changing as infants and young children are experiencing increasingly severe infections [7]. Numerous investigations have demonstrated that the majority of children acquire COVID-19 either via familial cluster transmission or intimate contact with an infected patient. Researchers detected viral shedding more than four weeks after the onset of the sickness, which raises serious concerns regarding COVID-19 fecal-oral transmission in children. Fecal-oral transmission is especially concerning in newborns and preschoolers who are not yet toilet trained [7]. The underlying rationale for COVID-19's lower incidence and milder symptoms in children is that a range of viral infections, particularly in younger children, have affected them. It is hypothesized that repeated viral exposure strengthens the immune system against SARS-CoV-2 infection. In addition, research has revealed that SARS-CoV-2 binds to the angiotensin-converting enzyme (ACE), which is underdeveloped in children, resulting in a decreased risk of SARS-CoV-2 infection [9]. Children with COVID-19 and a severe or life-threatening infection of the lower respiratory tract are typically hospitalized. Children with mild COVID-19 may require hospitalization if they are at risk for serious illness due to underlying conditions or if they are febrile neonates younger than 30 days old [10].

One of the most important measures of a hospital's operational effectiveness is the duration of stay, and hospital management and other associated professionals are particularly concerned about this metric. Excessively extended stays are a warning sign of a low bed turnover rate. In addition, there will be limits placed on the overall number of patients who can be treated. To this day, very few studies have been carried out to investigate and get an understanding of the factors that influence the length of time COVID-19 patients remain hospitalized. It will be highly advantageous for healthcare practitioners to understand these components to raise the rate of bed turnover, particularly during times of high healthcare demand when medical resources are constrained and scarce, such as during a pandemic [11]. The aforementioned performance indicator is dependent on factors that affect the delivery of healthcare, such as the number of available hospital beds, the types of payments that are accepted, and the policies that govern hospital discharges. It is also dependent on factors that affect the demand for health services, such as the severity of an illness, the direct and indirect costs associated with it, and the presence of other comorbid illnesses [12].

According to several national organizations, all pediatric patients with COVID-19 should get supportive care. Even in the most severe cases, most children with COVID-19 infection respond well to supportive care [13]. Among the routine supportive care measures are respiratory care, including supplementary oxygen and ventilatory support *noninvasive or invasive*; support for fluids and electrolytes; administration of empiric antibiotics as indicated for community-acquired or healthcare-associated pneumonia. The maintenance of empiric antibiotics should be evaluated by cultures and other microbiological testing, as well as the clinical state. Monitoring for cytokine release syndrome includes checking blood pressure for hypotension, oxygen saturation for worsening hypoxemia, and biomarkers, including obtaining baseline C-reactive protein (CRP), D-dimer, ferritin, lactate dehydrogenase (LDH), and interleukin-6 (IL-6), and providing thromboprophylaxis [9]. Lung imaging has been proven to be vital for the early detection and treatment of those infected with COVID-19. Chest radiography (CXR) and chest computed tomography (CT) are the most often used lung imaging modalities nowadays [14]. In a meta-analysis done to describe COVID-19 lung imaging data in the pediatric population, it was found that ground glass appearance was the most prevalent COVID-19 lung CT imaging finding in pediatric patients. Ground glass appearance was found in 37.2% of patients. The presence of consolidations or pneumonic infiltrates was the second most prevalent lung imaging result, occurring in 22.3% of patients [15]. Dexamethasone is advised for COVID-19 patients who are receiving ventilator assistance or supplementary oxygen. If dexamethasone is unavailable, it is permissible to use alternative glucocorticoids at equivalent dosages, but the evidence supporting their use is less thorough than those for dexamethasone. In contrast, it is not recommended to use dexamethasone or other glucocorticoids to treat or prevent mild-to-moderate COVID-19 in non-oxygen-dependent individuals. These recommendations are mostly consistent with those of other professional and governmental organizations [16]. Individualized decisions regarding the use of antiviral medication must be taken into consideration of the disease's severity, clinical history, existing evidence of its efficacy, and any underlying conditions that may increase the risk for the progression of severity. The focus of trials on the effectiveness and safety of antiviral therapy has been on adults with severe lower respiratory tract illness with few studies and trials regarding their focus on the use of antivirals in COVID-19-positive children. Antiviral treatment is advised for children with confirmed severe or serious COVID-19 infection, despite the lack of evidence demonstrating its benefits. Antiviral medicine may be beneficial for children with mild or moderate COVID-19 illness who have an underlying concurrent medical condition that increases the risk of clinical complications [17].

Our study was conducted on all patients who were admitted to Queen Rania Al Abdullah Hospital for Children over 24 months (February 2020- February 2022), and there were no criteria to include or exclude any patients.

## Materials and methods

We collected data over two years (February 2020-February 2022) for 100 COVID-19-positive patients who were admitted to Queen Rania Al Abdullah Hospital for Children, Amman. All patients admitted during the period mentioned were included, and no criteria to exclude or include any. We depended on a kit of COVID-19 polymerase chain reaction (PCR) nasal swabs to label patients positive for COVID-19 infection. The PCR kits were not specific for variants of COVID-19. We categorized the data according to gender, symptoms they had, signs, laboratory data findings, imaging findings, type of respiratory support, usage or no usage of corticosteroids, use of antiviral drugs, having or not having comorbidities, and the duration of hospitalizing each patient. We did not analyze comorbidities in the disease in itself due to the infrequency of that disease. For example, one had all infected with COVID-19 and hospitalized, another one had chronic kidney disease, other cystic fibrosis, etc. To be accurate if the disease per se increase or decreases the duration of stay, it must be frequent in number. We analyzed the data using SPSS BV Version 27 (IBM Corp., Armonk, NY, USA). Symptoms vary from asymptomatic to fever and respiratory illnesses, including cough, respiratory distress or cyanosis, fever, and gastrointestinal symptoms (GI) such as changes in bowel motion frequency and consistency, abdominal pain, or vomiting, fever, and central nervous symptoms (CNS) such as hallucination, change in the level of consciousness, change in activity pattern or headache, and blanching or non-blanching skin rashes. Signs were categorized as not having signs, abnormal air entry to the lungs, abnormal sound finding in the auscultating chest, respiratory distress signs, desatting, tachypnea, retraction, or pallor. Laboratory data vary from normal findings to leukocytosis, change in the differential of white blood cell (WBC) counts positive cultures of blood or urine, abnormality in kidney function test findings, abnormal liver enzymes, abnormalities in coagulation pattern, blood gases abnormalities, or high erythrocyte sedimentation rate (ESR). The imaging findings vary from normal findings to patchy consolidation on CXR, finding pleural effusion using chest ultrasound, or discovering consolidations using a chest CT scan. Hospitalized COVID-19 patients were admitted to Queen Rania Al Abdullah Hospital for Children in isolated wards and/or pediatrics intensive care unit (PICU). Queen Rania Al Abdullah Hospital for Children is the first specialized medical center for children in Jordan that is distinguished by modern, advanced, and comprehensive healthcare services. It is one of the Health Care Accreditation Council (HCAC) accredited hospitals.

## Results

Of the 100 patients, using frequency descriptive statistics, 53 were males and 47 were females. The maximum length of stay (LOS) at the hospital recorded was 28 days, while the minimum was one day. Five days was the most frequent LOS among patients, reaching 21%. Seven patients needed intensive care admission. Of the total number of patients, 67% used steroids and 33% did not. Fifty-two percent of the patients had associated chronic illnesses, while 48% were medically free. Eighty-nine percent of the patients did not receive antiviral drugs, 4% were prescribed Sancovir, and 7% remdesivir. Eighty-five percent of the patients did not need respiratory support, seven needed nasal cannula, four needed mechanical ventilator, three needed Vapotherm, and one patient needed a face mask. Using mean descriptive statistics, we compared LOS and having or not having comorbidities. The mean LOS of patients with a comorbid disease was 6.15 days, and the maximum was 28 days, while the mean LOS was 4.81 days in patients without chronic illnesses and a maximum of 14 days (Table 1).

**Table 1:** Length of stay in comorbid patients versus medically free patients. *N*, number of patients

Comorbidity	Mean	N	Standard Deviation	Median	Minimum days	Maximum days	% of total sum	% of total *N*	Range	Standard error of the mean	
No comorbidity	4.81	52	2.716	4	1	14	45.90	52	13	0.377	
Associated comorbidity	6.15	48	4.515	5	2	28	54.10	48	26	0.652	
Total	5.45	100	3.732	5	1	28	100	100	27	0.373	

After using the independent sample t-test analysis, the significance was 0.07 (Table 2).

**Table 2:** Significance of associated comorbidity on the length of stay. df, degrees of freedom

Independent samples test									
	Levene's test for equality of variances		*t*-test for equality of means						
	F	Sig.	t	df	Sig. (two-tailed)	Mean difference	Standard error difference	95% confidence interval of the difference	
								Lower	Upper
Length of stay	2.136	0.147	-1.812	98	0.073	-1.338	0.739	-2.804	0.127
			-1.778	75.839	0.079	-1.338	0.753	-2.837	0.161

The average LOS for patients who used steroid was 4.9 days, while those who did not use steroid was 6.5 days, with a significance of 0.04. A long LOS of 12 days was recorded in patients with abnormal chest CT scan, and a short LOS of two days was reported in patients with minimal pleural effusion on ultrasound with significance 0.01. Fifteen days duration was the mean LOS for patients used mechanical ventilator, while the shortest LOS recorded of one day in patients who needed only a face mask with a significance of 0.000. The maximum day duration was 14 days for patients who had respiratory symptoms recording mean of 7.08 of patients, and the minimum was one day for patients having fever and respiratory symptom with significance of 0.8. In regards to respiratory symptoms, 20 days was the maximum duration recorded in patients having respiratory distress while the minimum was one day for patient with stridor; the significance was 0.16. Regarding laboratory results and their correlation to LOS, 28 days was the maximum stay for patients with pancytopenia, while the LOS was one day in patients having normal lab findings; the significance was 0.000. In cross-tabulations, the correlation between previous health status and imaging reports shows 38% who reported normal study were previously healthy and 33% had comorbidities. Four patients had perihilar infiltrates and comorbid chronic illness (Table 3).

**Table 3:** Imaging studies in comorbid patients. CT, computer tomography; U/S, ultrasound

		Comorbidity		Total
		No comorbidity	Associated comorbidity	
Imaging study results	Normal	38	33	71
	Bilateral patchy consolidation	2	0	2
	Chest CT scan: multiple consolidations in both lungs	0	1	1
	Bilateral perihilar infiltrates	3	4	7
	Diffuse reticulonodular shadowing	3	3	6
	Bilateral broncopneumonia	2	1	3
	Metastatic lung lesions	0	1	1
	Chest U‎/S minimal left-sided pleural effusion	0	1	1
	Right-sided infiltration	1	3	4
	increased bronchovascular markings	3	1	4
Total		52	48	100

Of the total patients, 67% needed steroids and 33% did not. Of the 67% needing steroid, 33% were comorbid, while 34% were previously healthy (Tables 4-5).

**Table 4:** Use of steroids in comorbid patients/cross-tabulation.

		Use of steroids		Total
		Yes	No	
Comorbidities	No comorbidity	34	18	52
	Associated comorbidities	33	15	48
Total		67	33	100

**Table 5:** Use of steroids in comorbid patients (chi-square tests). df, degree of freedom

Chi-square tests	Value	df	Asymptotic significance (two-sided)	Exact sig. (two-sided)	Exact sig. (one-sided)
Pearson chi-square	0.128	1	0.721		
Continuity correction	0.021	1	0.885		
Likelihood ratio	0.128	1	0.721		
Fisher's exact test				0.832	0.443
Linear-by-linear association	0.127	1	0.722		
Number of valid cases	100				

The patients who stayed 28 days did not receive antiviral drugs, while those who stayed 20 days received remdesivir. Forty-five percent of the patients who did not use antiviral drugs were previously healthy, 3% received Sancovir, and 4% received remdesivir. On the other hand, 44% of the patients previously having comorbidity did not need antiviral drugs, 1% had Sancovir, and 3% needed remdesivir (Table 6).

**Table 6:** Use of antivirals in comorbid patients/cross-tabulation.

		Use of antiviral drugs			Total
		No	Sancovir	Remdesivir	
Comorbidity	No comorbidity	45	3	4	52	
	Associated comorbidity	44	1	3	48
Total		89	4	7	

Forty-four percent of previously healthy children did not need respiratory support. Four percent of healthy patients needed nasal cannula, while 3% who needed nasal cannula were having chronic illness (Table 7). Among our patients, no deaths were recorded.

**Table 7:** Respiratory support in comorbid patients/cross-tabulation.

		Respiratory support					Total
		No use	Nasal cannula	Mechanical ventilator	Face mask	Vapotherm	
Comorbidity	No comorbidity	44	4	2	0	2	52	
	Associated comorbidity	41	3	2	1	1	48
Total		85	7	4	1	3	100

## Discussion

Our study included only COVID-19-positive patients aged up to 14 years who were hospitalized at Queen Rania Al Abdullah Hospital for Children, a tertiary hospital and one of the military hospitals in Jordan. The 100 patients were admitted from February 2020 to February 2022, and there were no criteria to exclude or include any. The COVID-19 PCR kit was not specific for a variant. The reason behind the specificity is that all kits available at the beginning of the pandemic were used and the only relied on and available. We did not analyze the disease in itself influencing LOS due to its infrequency. For example, one had all infected with COVID-19 and hospitalized, another one had chronic kidney disease, the other had cystic fibrosis, the next one had Crohn's disease, etc. COVID-19 infection is a mild illness with a low death rate in children and adolescents. Higher admission rates are connected with fever, rash, and comorbidities. In the pediatrics group, age influences the clinical range and severity of infection. Continuous monitoring is required to better understand pediatric COVID-19 infection, guide treatment, and assess the need for immunization in children [18]. LOS is one of the efficiency and quality indicators being actively researched by medical academics to improve the quality and effectiveness of healthcare services [19]. The median LOS in our study was five days, which is close to the value published in a similar study in Europe, the United States, Italy, the United Kingdom, and China [20]. In the data analysis provided to the Latin-American Society of Pediatric Infectious Diseases (SLIPE-COVID) research network in Latin America to evaluate parameters impacting admission to wards and PICUs among COVID-19-positive pediatrics, it was determined that 47% were hospitalized, 84% to the pediatric wards, and 16% to the PICU [21]. In this study, of the 100 hospitalized patients, 7% needed PICU resources. Numerous randomized trials appear to suggest that systemic corticosteroid treatment positively affects health outcomes and decreases mortality in hospitalized COVID-19-positive patients who need supplemental oxygen. In comparison, systemic corticosteroids have not been demonstrated to help or impair hospitalized COVID-19-positive patients who do not require supplementary oxygen [22]. The majority of COVID-19 patients are treated symptomatically. In clinical practice, corticosteroids are frequently used for the symptomatic treatment of severe viral pneumonia. However, it is still controversial whether COVID-19 individuals should be treated with corticosteroids [23]. A meta-analysis carried out to assess the percentage and effectiveness of systemic corticosteroid treatment for COVID-19 patients shows that the proportion of COVID-19 patients who received corticosteroids was significantly lower than the proportion of COVID-19 patients who did not receive corticosteroids (35.19% vs. 64.49%). The systematic review concludes that early use of a short course of Methylprednisolone, an inexpensive and readily available agent, in patients with moderate-to-severe COVID-19 infection may be beneficial [24]. Our findings reveal that 67% needed corticosteroids, while 33% did not. The average LOS for those who used steroids was 4.9 days, while those who did not use steroids stayed for 6.5 days. A meta-analysis was done regarding the use of antivirals in treatment regimens of COVID-19 in the pediatric age group, and it showed that antivirals were administered in 15.3% of children, and remdesivir was the most often used antiviral medicine in 6.2% of included children, with no major side effects [25]. Recent studies have indicated that remdesivir should be used only in severe situations, even in adults. Randomized evaluation of the COVID-19 Therapy (Recovery) trial found that while it lowers intensive care unit stay, it does not reduce mortality [26]. Our results show that 89% of patients did not receive antiviral drugs and their LOS was eight days longer than patients who received remdesivir as an antiviral therapy. Compared to adults, pediatric COVID-19 infection is generally milder; a few of these children become seriously ill and require respiratory support. In observational research done on COVID-19 pediatric inpatients to determine variables related to unfavorable short-term outcomes, it was shown that 35.5% needed respiratory assistance during their hospitalization, resulting in a lengthened hospital stay of over seven days [27]. Our results show that 85% of patients did not need respiratory assistance (e.g., nasal cannula, mechanical ventilation, and Vapotherm), and only 15% needed respiratory support with 15 days as the mean of hospital stay in patients using a mechanical ventilator, and the least LOS was recorded in those using a face mask. Findings in the literature support the notion that the existence of the concomitant disease in pediatric COVID-19 inpatients is uncorrelated with the LOS. A study done in an Indian tertiary children hospital showed that having comorbidities did not affect the severity of the illness, length of hospitalization, ventilation demand, or death [28]. In our analysis, we did not identify the underlying comorbidities, and all individuals with any medical history were included in this category. Forty-eight percent of patients had associated comorbidities, and their mean LOS was 6.1 days, while for patients with no past health issues, the mean LOS was 4.8 days, and the significance value was >0.05. Lymphocytopenia, which is a common sign of adult COVID-19 and is thought to be one of the indications of disease severity, was uncommon in children with COVID-19. Previous research has demonstrated that SARS-CoV-2 causes a sequence of immunological reactions after entering the body, which subsequently causes inflammatory storms, resulting in an increase in inflammatory markers and a decrease in lymphocyte numbers. Low WBC count, elevated CRP values, elevated ESR, and elevated ALT values were recorded less frequently in children, indicating that children with COVID-19 illness had inadequate immunological responses [29]. Our study shows that having normal laboratory findings had a minimum LOS of one day. The imaging results in the aforementioned research revealed that around 36% of children's chest CT scans had no imaging abnormalities, indicating that children with COVID-19 had only minor local invasion [29]. In our sample, the patients who had abnormal chest CT findings stayed in the hospital for a mean of 12 days, and the most common finding was bilateral perihilar infiltrates.

## Conclusions

Children with COVID-19 are mostly asymptomatic or have very minor symptoms, and serious disease in children is uncommon. A minority of children experience severe symptoms, necessitating hospitalization to the ward or the PICU. The LOS is an essential indication of hospital management efficiency. Understanding the factors affecting an extended LOS is an important key to reducing the number of inpatient days, thereby reducing the risk of adverse effects and improving treatment quality. In our analysis, we found that the existence of a comorbid illness in pediatric hospitalized patients with COVID-19 had no effect on LOS in hospital, while using steroids had a significant effect on shortening LOS. Another significant result of our study was the correlation found between abnormal findings in CT scans, which was found to result in an increased LOS. Having or not having severe respiratory symptoms did not influence the LOS. The aforementioned results in this study did not specify any variants of COVID-19 infection. In our upcoming studies, we will aim to specify those variants and try to understand whether any of those variants have influenced the severity of illness and LOS of hospitalized patients.
